# Chrysin and Luteolin from Moroccan Propolis to Prevent Aggressive Periodontitis Caused by *Aggregatibacter actinomycetemcomitans* Leukotoxin: A Computer-Aided Drug Design Approach

**DOI:** 10.3390/ph19010115

**Published:** 2026-01-08

**Authors:** Doha EL Meskini, Fettouma Chraa, Jihane Touhtouh, Mouna Ouadghiri, Monica Gallo, Abdelhakim Bouyahya, Tarik Aanniz

**Affiliations:** 1 Medical Biotechnology Laboratory (MedBiotech), Bioinova Research Center, Medical and Pharmacy School, Mohammed V University in Rabat, Rabat 10100, Morocco; elmeskinidoha2@gmail.com (D.E.M.); fettoumachraa04@gmail.com (F.C.); m.ouadghiri@um5r.ac.ma (M.O.); 2 Laboratory of Natural Resources and Environment, Polydisciplinary Faculty of Taza, Sidi Mohamed Ben Abdellah University of Fez, Taza 30050, Morocco; jihanetou97@gmail.com; 3 Department of Molecular Medicine and Medical Biotechnology, University of Naples Federico II, Via Pansini 5, 80131 Naples, Italy; 4 Laboratory of Human Pathologies Biology, Faculty of Sciences, Mohammed Vth University in Rabat, Rabat 10100, Morocco; a.bouyahya@um5r.ac.ma

**Keywords:** *A. actinomycetemcomitans*, leukotoxin, aggressive periodontitis, chrysin, luteolin, MD simulations

## Abstract

**Background**: *Aggregatibacter actinomycetemcomitans* is a Gram-negative, facultative anaerobic, immobile oral bacterium responsible for the secretion of virulence factors, namely leukotoxin (LtxA), a large exotoxin of the RTX family that enables the bacterium to evade the immune system by destroying leukocytes, resulting in aggressive periodontitis (AP) leading to tooth loss. **Methods**: This study aimed to screen 106 molecules derived from Moroccan propolis in order to identify potential inhibitors of the active sites of LtxA based on molecular docking, ADMET property evaluation, and molecular dynamics (MD) simulation. **Results**: Epigallocatechin gallate (EGCg), used as a reference compound, showed binding energies of −6.9 kcal/mol, −6.1 kcal/mol, −6.5 kcal/mol, and −5.9 kcal/mol with the four active sites P1, P2, P3, and P4, respectively. By establishing conventional hydrogen bonds, pi-alkyl bonds, and non-covalent pi–pi bonds. Chrysin and luteolin showed favorable binding affinities with the four active sites, named as follows: P1–P4 (P1–chrysin = −7.5 kcal/mol; P2–chrysin = −7.9 kcal/mol; P3–chrysin = −8.1 kcal/mol; P4–chrysin = −6.9 kcal/mol; P1–luteolin = −7.3 kcal/mol; P2–luteolin = −7.6 kcal/mol; P3–luteolin = −8.1 kcal/mol; P4–luteolin = −7.3 kcal/mol). The binding affinity of these two propolis derivatives was stabilized by pi−sigma bonds, pi−alkyl bonds, conventional hydrogen bonds, pi-cation interactions, non-covalent pi–pi bonds, and carbon–hydrogen bonds. According to free energy calculations performed with Prime MM-GBSA, the complexes formed by chrysin demonstrated the most stable interactions due to Van der Waals and lipophilic forces. Luteolin formed significant interactions, but slightly weaker than those of chrysin. These results reveal the inhibitory potential of chrysin and luteolin with protein active sites. MD simulations corroborated the excellent stability of complexes formed by chrysin, as indicated by low RMSD values, suggesting favorable dynamic behavior. **Conclusions**: These results highlight the potential of chrysin as a versatile inhibitor capable of interacting with the four active sites. These findings are a strong foundation for further experimental confirmations.

## 1. Introduction

Periodontitis is an inflammatory disease of infectious origin that affects more than 40% of adults [1]. It specifically targets the periodontium, that is, the tissues supporting the teeth, which can lead to their loss [2]. It first manifests as chronic inflammation of the gingiva, then gradually progresses until it reaches the alveolar bone [2,3]. Individuals with rheumatoid arthritis—a chronic inflammatory disease that attacks the joints—as well as those exposed to smoking, having poor oral hygiene, or experiencing stress, are at an increased risk of developing this infection [2,4]. In this context, it is essential to focus on the etiological agent responsible for this pathology, which was discovered in 1976 [5]—previously known as *Actinobacillus actinomycetemcomitans*, and, since 2006, named *Aggregatibacter actinomycetemcomitans* (*A. actinomycetemcomitans*) [6,7].

*A. actinomycetemcomitans* is a Gram-negative, anaerobic, and non-motile bacterium [6] found in the oral cavity of a large portion of the human population [8]; it is responsible for aggressive periodontitis (AP), which primarily affects healthy young individuals [2,9,10]. Hence, to better understand the virulence of *A. actinomycetemcomitans* in AP, it is essential to examine its virulence factors, which confer its infectious potential—namely biofilm polysaccharides, lipopolysaccharides (LPSs), and toxins [11]. This bacterium generates two types of toxins, namely the cytolethal distension toxin (CDT) and leukotoxin (LtxA), which help *A. actinomycetemcomitans* escape and evade the immune system [11,12]. The peculiarity is that CDT and LtxA are produced distinctly and have different immune targets [9,11]. Indeed, CDT inhibits cell proliferation, leading to their death, while LtxA specifically targets blood cells (hematopoietic cells) [2].

Here, we primarily focused on LtxA due to its marked virulence [9,11]. LtxA is a large toxin belonging to the RTX (repeat-in-toxin) family. It is composed of 1055 amino acids and is formed of four domains. The first domain, the N-terminal, is formed by amphipathic helices and the CRAC motif (Cholesterol Recognition/Interaction Amino Acid Consensus), which plays a role in the interaction with cholesterol in cell membranes. The second domain, the central domain, is common to all RTX toxins and activates LtxA. The third domain is formed by a repetition of the motif (glycine–glycine), characteristic of the RTX family. Finally, the fourth domain, the C-terminal, contributes to the exposure of the toxin outside the bacterium [9]. This leukotoxin has approximately 40% sequence homology with *Mannheimia haemolytica LktA* and 50% with *Escherichia coli HlyA* [9,13,14]. In its free form, LtxA can interact with leukocytes by binding either to cholesterol or to the LFA-1 receptor (Lymphocyte Function-Associated Antigen-1), found on the surface of leukocytes. The interaction leads to a complete collapse of the cell membrane, followed by the internalization of the toxin, ultimately resulting in cell death (cell necrosis). In addition, in its associated form, LtxA can be transported by bacterial outer membrane vesicles (OMVs), where it still retains its activity against host cells (leukocytes). In this situation, the interaction depends neither on cholesterol nor on LFA-1 [15]. Computer-Aided Drug Design (CADD) studies are based on the exploration of databases, the search for similarities, structure–activity relationships, computational modeling, pharmacophore identification, molecular docking and dynamics, high-throughput screening, and others [16,17,18,19,20]. In the context of this study, we attempted to identify potential lead compounds against LtxA compared to the reference molecule epigallocatechin gallate (EGCg) to prevent AP using virtual screening of biomolecules derived from Moroccan propolis [10,15]. The fundamental idea behind these drugs and bioactive molecules lies in their interaction affinity with protein targets [2,21].

Propolis is a resinous substance naturally formed by bees. It is made up of wax, enzymes from bee saliva, buds, and secretions from various plants and trees [22]. It plays a role as a protective barrier against pathogens and as a collective defense for the bee colony [23]. It possesses several pharmaceutical properties, including antibacterial, antifungal, antidiabetic, and anticancer, which explains its long-standing use as a traditional remedy for human activities [24,25,26]. However, the molecular processes and underlying mechanism of action remain poorly understood [24,25,27]. The identified compounds of propolis are classified into distinct categories, namely flavonoids, phenols, terpenoids, fatty acids, aromatic acids, volatile oils, and amino acids [28].

Here, we performed a screening of 106 propolis-derived compounds on the four active sites of the LtxA since P1 and P2 are two active sites of the N-terminal region of LtxA, involved in attachment to the cell membrane via the CRAC motif, while P3 and P4 are two active sites of the RTX region of LtxA, involved in binding to the LFA-1 receptor [9]. Hence, by targeting these four potential areas, the toxicity caused by *A. actinomycetemcomitans* could be altered.

## 2. Results

Finding effective treatments for LtxA remains a complicated task. Natural products derived from propolis represent a promising avenue as they may be more effective and safer than synthetic drugs [29]. Many studies are currently beginning with in silico analyses, providing important fundamental information about candidate molecules. These results are then used as a basis for more advanced and rational in vitro and in vivo testing [30].

### 2.1. Virtual Screening and Molecular Interaction Studies

EGCg is used as a reference for docking [15] and showed binding energies of −6.9 kcal/mol, −6.1 kcal/mol, −6.5 kcal/mol, and −5.9 kcal/mol with the four active sites P1, P2, P3, and P4, respectively. The two best molecules targeting these four active sites were chrysin (5,7-dihydroxyflavone) and luteolin (3′,4′,5,7-tetrahydroxyflavone). For the same four sites, chrysin showed binding energies of −7.5 kcal/mol, −7.9 kcal/mol, −8.1 kcal/mol, and −6.9 kcal/mol, respectively, while luteolin exhibited binding energies of −7.3 kcal/mol, −7.6 kcal/mol, −8.1 kcal/mol, and −7.3 kcal/mol, respectively. This makes both molecules the best candidates to target the LtxA toxin.

**Figure 1:**
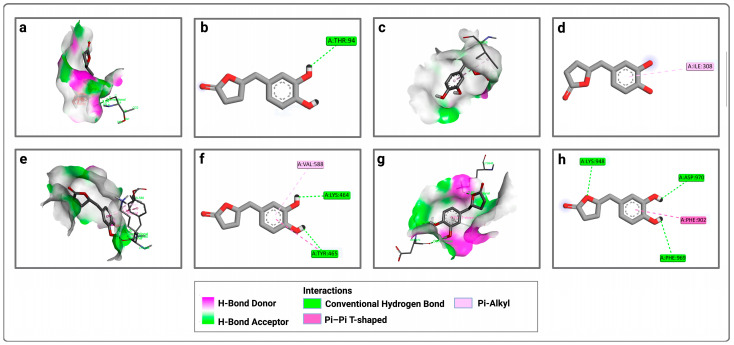
Representation of the interactions of EGCg with the four active sites of LtxA: (**a**) 3D visualization of interactions within the EGCg–P1 complex; (**b**) 2D visualization of interactions within the EGCg–P1 complex; (**c**) 3D visualization of interactions within the EGCg–P2 complex; (**d**) 2D visualization of interactions within the EGCg–P2 complex; (**e**) 3D visualization of interactions within the EGCg–P3 complex; (**f**) 2D visualization of interactions within the EGCg–P3 complex; (**g**) 3D visualization of interactions within the EGCg–P4 complex; (**h**) 2D visualization of interactions within the EGCg–P4 complex.

**Figure 2:**
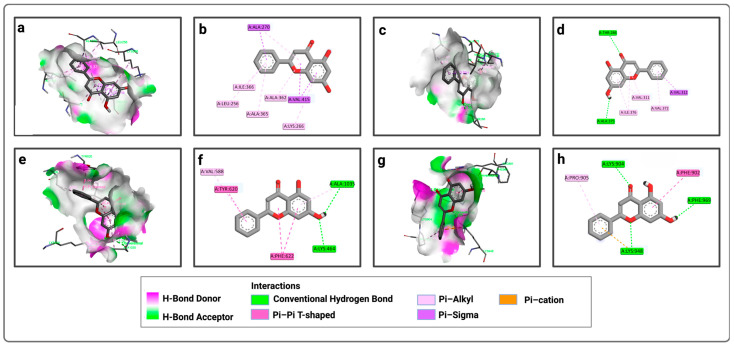
Representation of the interactions of chrysin with the four active sites of LtxA: (**a**) 3D visualization of interactions within the chrysin–P1 complex; (**b**) 2D visualization of interactions within the chrysin–P1 complex; (**c**) 3D visualization of interactions within the chrysin–P2 complex; (**d**) 2D visualization of interactions within the chrysin–P2 complex; (**e**) 3D visualization of interactions within the chrysin–P3 complex; (**f**) 2D visualization of interactions within the chrysin–P3 complex; (**g**) 3D visualization of interactions within the chrysin–P4 complex; (**h**) 2D visualization of interactions within the chrysin–P4 complex.

**Figure 3:**
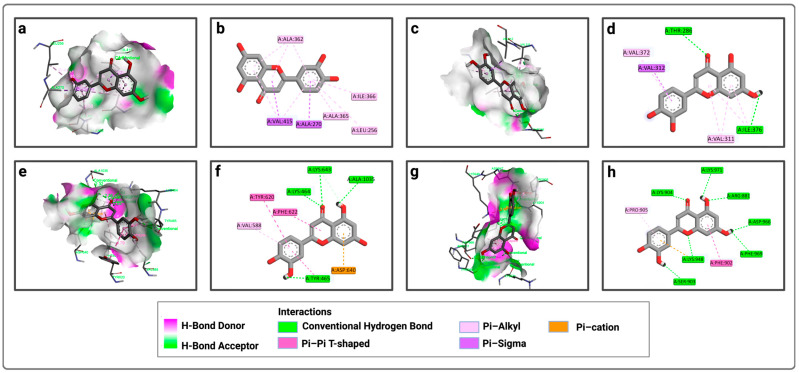
Representation of the interactions of luteolin with the four active sites of LtxA: (**a**) 3D visualization of interactions within the luteolin–P1 complex; (**b**) 2D visualization of interactions within the luteolin–P1 complex; (**c**) 3D visualization of interactions within the luteolin–P2 complex; (**d**) 2D visualization of interactions within the luteolin–P2 complex; (**e**) 3D visualization of interactions within the luteolin–P3 complex; (**f**) 2D visualization of interactions within the luteolin–P3 complex; (**g**) 3D visualization of interactions within the luteolin–P4 complex; (**h**) 2D visualization of interactions within the luteolin–P4 complex.

Then, the analysis of the interactions of both ligands with the four active sites was performed to understand the obtained results (Figure 1, Figure 2 and Figure 3). At the P1 active site, EGCg forms only a conventional hydrogen bond with THR 94 (Figure 1a,b). At the second active site (P2), it forms a pi–alkyl bond with ILE 308 (Figure 1c,d). For the third active site (P3), EGCg forms conventional hydrogen bonds with TYR 465 and LYS 464, a pi-alkyl bond with VAL 588, and a non-covalent pi–pi bond with TYR 465 (Figure 1e,f). For the fourth active site (P4), EGCg forms conventional hydrogen bonds with LYS 948, ASP 970, and PHE 969, and a pi–pi bond with PHE 902 (Figure 1g,h).

For P1, chrysin forms pi–sigma bonds with amino acids ILE 366, LEU 256, ALA 365, ALA 362, and LYS 266, and pi–alkyl bonds with VAL 415 and ALA 270 (Figure 2a,b). For the second active site (P2), chrysin forms conventional hydrogen bonds with ALA 375 and THR 286, a pi–sigma bond only with VAL 312, and pi–alkyl bonds with VAL 311, VAL 372, and ILE 376 (Figure 2c,d). For the third active site, chrysin forms conventional hydrogen bonds with ALA 1035 and LYS 464, a pi–alkyl bond with VAL 588, and non-covalent pi–pi bonds with PHE 622 and TYR 620 (Figure 2e,f). For the fourth active site, chrysin forms conventional hydrogen bonds with LYS 904, PHE 969, and LYS 948, a pi–cation interaction with LYS 948, a pi–pi bond with PHE 902, and a pi-alkyl bond with PRO 905 (Figure 2g,h).

Similarly, for P1, luteolin forms pi–sigma bonds with VAL 415 and ALA 270, and pi–alkyl bonds with ALA 365, LEU 256, ILE 366, and ALA 362 (Figure 3a,b). For P2, luteolin forms conventional hydrogen bonds with THR 286 and ILE 376, a pi–sigma bond only with VAL 312, pi-alkyl bonds with VAL 372 and VAL 311, and carbon–hydrogen bonds with ILE 376 (Figure 3c,d). For P3, luteolin forms conventional hydrogen bonds with LYS 464, LYS 643, ALA 1035, and TYR 465, a carbon–hydrogen bond only with LYS 643, a pi–anion bond with ASP 640, pi–pi bonds with TYR 620 and PHE 622, and a pi–alkyl bond with VAL 588 (Figure 3e,f). Meanwhile, for P4, luteolin forms conventional hydrogen bonds with LYS 904, LYS 971, ARG 881, ASP 966, PHE 969, LYS 948, and SER 903, a pi–cation interaction with LYS 948, a pi–pi bond with PHE 902, and a pi–alkyl bond with PRO 905 (Figure 3g,h).

Based on the docking results and interactions analysis, we conclude that the two bioactive molecules bind effectively to the four active sites by establishing hydrophobic, electrostatic, non-covalent interactions and hydrogen bonds with different protein residues.

### 2.2. Prediction of ADME-Tox Properties

The in silico prediction of ADMET-Toxicity results for chrysin and luteolin are shown in Table 1. Examination of the physicochemical characteristics reveals that the two compounds studied complied with Lipinski’s rules, confirming their high oral bioavailability [31]. The results reveal that both compounds are characterized by good skin permeability, exceeding a logKp value of −2.5 [32], and show very high intestinal absorption above 30% [33], indicating good oral bioavailability and excellent intestinal absorption after oral administration [31]. They are characterized by moderate water solubility, except that luteolin is more soluble than chrysin.

Chrysin and luteolin are characterized by low Caco2 permeability, which is 0.945 × 10^−6^ cm/s and 0.096 × 10^−6^ cm/s, respectively (values below 8 × 10^−6^) [34]. However, predictions of blood–brain barrier (BBB) permeability have shown that both molecules cross the BBB very poorly, with logBB values below −1 [35,36]. The compounds are neither substrates nor inhibitors of major CYP enzymes, such as CYP2D6 and CYP3A4, suggesting a low risk of interactions with other drugs.

The toxicity profile is favorable for both compounds, with no signs of AMES toxicity (absence of genetic mutation) [37], no hepatotoxicity, and no inhibitory effect on hERG I and II potassium channels, which are essential for heart rhythm [37]. According to the maximum tolerated dose reference threshold of 0.477, chrysin has a maximum tolerated dose of 0.016 (log mg/kg/day), which is lower than that of luteolin at 0.499 log mg/kg/day, indicating that chrysin exhibits toxicity more rapidly than luteolin.

### 2.3. MD Simulations and Bond Energy Calculations

#### 2.3.1. MD Simulations of P1 Complexes

RMSD is one of the parameters that studies atomic movements during a simulation period based on a reference structure, which determines the stability of the complex during the simulation [38,39]. Root mean square fluctuation (RMSF) measures the displacement of protein residues relative to a mean position when the ligand binds to the active site during a simulation period [39]. Structural stability is reflected in low RMSF values, indicating reduced atomic fluctuations [40].

**Figure 4:**
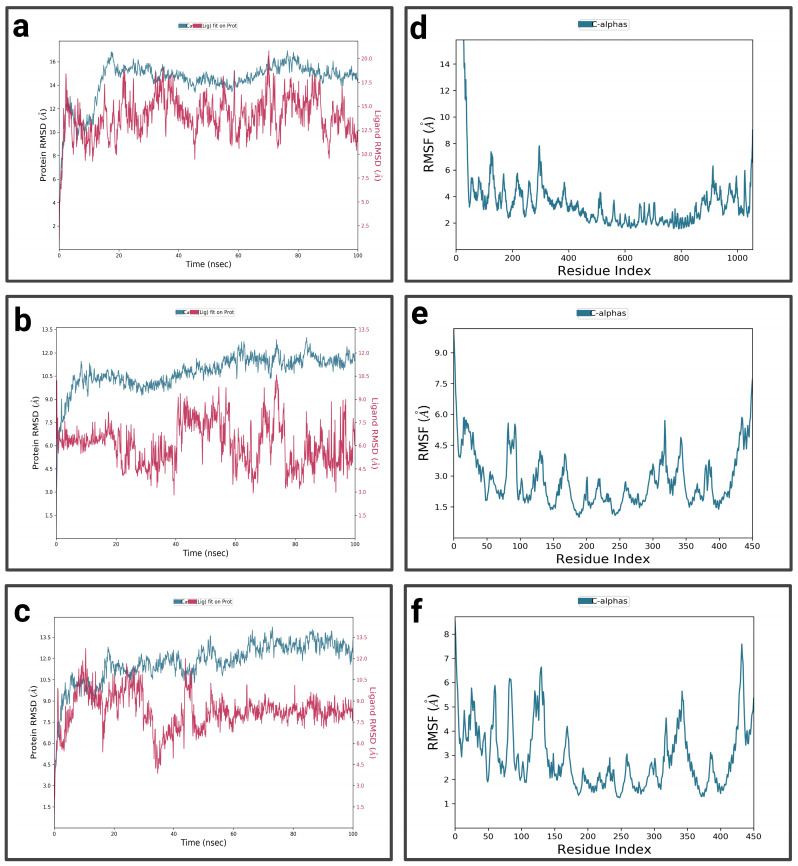
Analysis of molecular dynamics simulation: (**a**) RMSD of the P1-EGCg complex; (**b**) RMSD of the P1–chrysin complex; (**c**) RMSD of the P1–luteolin complex; (**d**) RMSF of the P1-EGCg complex; (**e**) RMSF of the P1–chrysin complex; (**f**) RMSF of the P1–luteolin complex.

For P1-EGCg (Figure 4a), during the first 15 nanoseconds, the protein exhibits significant fluctuations, indicating structural changes. It then stabilizes around 14.5 Å. The ligand is characterized by an increase in RMSD at the beginning of the simulation, reaching 18.75 Å—indicating significant conformational changes at the P1 active site—then its RMSD fluctuates between 12.5 Å and 15 Å during the rest of the simulation, suggesting a dynamic interaction pattern within the active site. The P1-EGCg complex shows high flexibility around residues 100, 200, and 1000 (RMSF between 6 Å and 14 Å) (Figure 4d). Residues located in the 400–800 range are relatively stable, characterized by RMSF values below 4 Å. For the P1–chrysin complex (Figure 4b), in the first 15 ns of the simulation, the protein exhibits fluctuations, indicating structural changes. It then stabilizes around 9.5 Å, with small changes, suggesting convergence toward a relatively equilibrated conformation. Meanwhile, the ligand increases at the beginning of the simulation, reaching a peak of 10 Å, indicating its conformational adaptation at the P1 level. It then presents fluctuations between 6 and 7.5 Å. The P1–chrysin profile shows that most residues are characterized by minor fluctuations of less than 4.5 Å, indicating structural stability, with the exception of a few peaks reaching 6 Å around residues 5 and 325 (Figure 4e). For P1–luteolin (Figure 4c), in the first 10 ns of the simulation, the RMSD of the protein gradually increases to 10.5 Å, then stabilizes around 11.25 Å with slight fluctuations for the remainder of the simulation, reflecting an equilibrated yet flexible protein conformation. In the first 40 ns, the ligand exhibits pronounced variations, probably due to conformational changes at the P1 active site, then shows moderate stabilization around 8.25 Å for the rest of the simulation, while exhibiting slight orientations at P1. The RMSF profile of the P1–luteolin complex reveals significant fluctuations exceeding 5 Å for most protein residues (Figure 4f), except for residues around 100 and 250, showing fluctuations of less than 3 Å.

**Figure 5:**
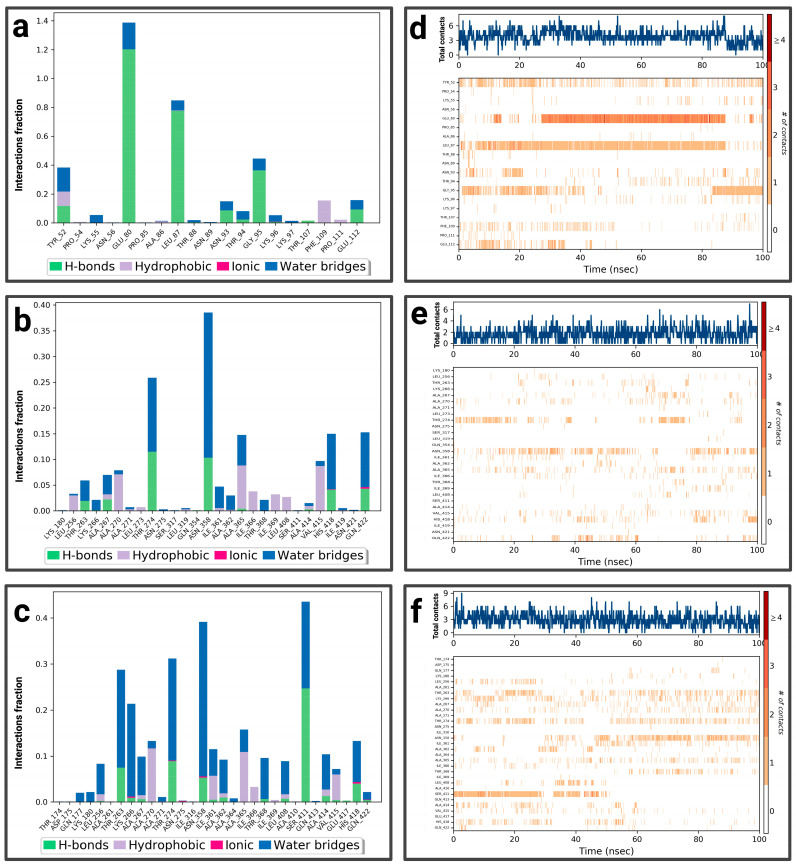
Analysis of molecular dynamics simulation: (**a**) histogram of P1-EGCg interactions; (**b**) histogram of P1–chrysin interactions; (**c**) histogram of P1–luteolin interactions; (**d**) P1-EGCg contact timeline; (**e**) P1–chrysin contact timeline; (**f**) P1–luteolin contact timeline.

For the P1-EGCg complex, GLU 80 represents the highest fraction percentage (1.4) formed by a water bridge and a hydrogen bond, revealing its significant role in stabilizing the ligand (Figure 5a). LEU 87 (0.8) and GLY 95 (0.4) establish the same type of interactions. TYR 52 (with a fraction of 0.39) forms a hydrogen bond overridden by a water bridge. Figure 5d illustrates the contact chronology of the P1-EGCg complex. The upper plot reveals that the total number of contacts varies between 2 and 3 over time, suggesting a stable interaction. The residues TYR 52, GLU 80, GLY 95, and LEU 87 establish persistent contacts during the simulation. For chrysin (Figure 5b), interactions were established with ASN 358, representing the highest fractional percentage (0.37), formed by a hydrogen bond and a water bond. THR 274 (fraction ≈ 0.25) and HIS 418 (≈0.15) form the same type of interactions. GLN 422 reaches a fractional value around 0.152, formed by a hydrogen bond and ionic bond, which are overcome by a water bridge. ALA 365 and VAL 415 form hydrophobic bonds, topped by a water bridge with fractions of ≈ 0.149 and 0.10, respectively. The total number of contacts varies between 2 and 6, with considerable stabilization after 20 ns. The amino acids ASN 358, HIS 418, GLN 422, ALA 365, and VAL 415 establish regular contacts (2 to 4)—illustrated by orange coloring—throughout the simulation (Figure 5e). For the P1–luteolin complex (Figure 5c), SER 411 represents the highest interaction percentage (greater than 0.4), with THR 263 (fraction 0.35) formed by a hydrogen bond topped by a water bridge. THR 368, with a respective fraction index of 0.09, establishes the same types of interactions. ASN 358 (fraction 0.39), THR 274 (fraction 0.3), and LYS 266 (fraction 0.2) establish three types of hydrogen and ionic bonds topped by a water bridge. ALA 267 (fraction 0.09) forms three types of hydrogen interactions, hydrophobic and hydric bonds. ALA 270 (fraction 0.12) and ALA 365 (fraction 0.15) form two types of hydrophobic interactions topped by a water bridge. According to Figure 5f, the contact chronologies of the P1–luteolin complex persisted from 40 ns onwards, with a number of contacts between 2 and 6. The following amino acids, THR 263, THR 368, ASN 358, THR 274, LYS 266, ALA 267, ALA 270, and ALA 365, established regular contacts (2–4)—illustrated by a dark orange color—throughout the simulation. More details about the residue-level contact are provided in the Supplementary Materials.

#### 2.3.2. MD Simulations of P2 Complexes

**Figure 6:**
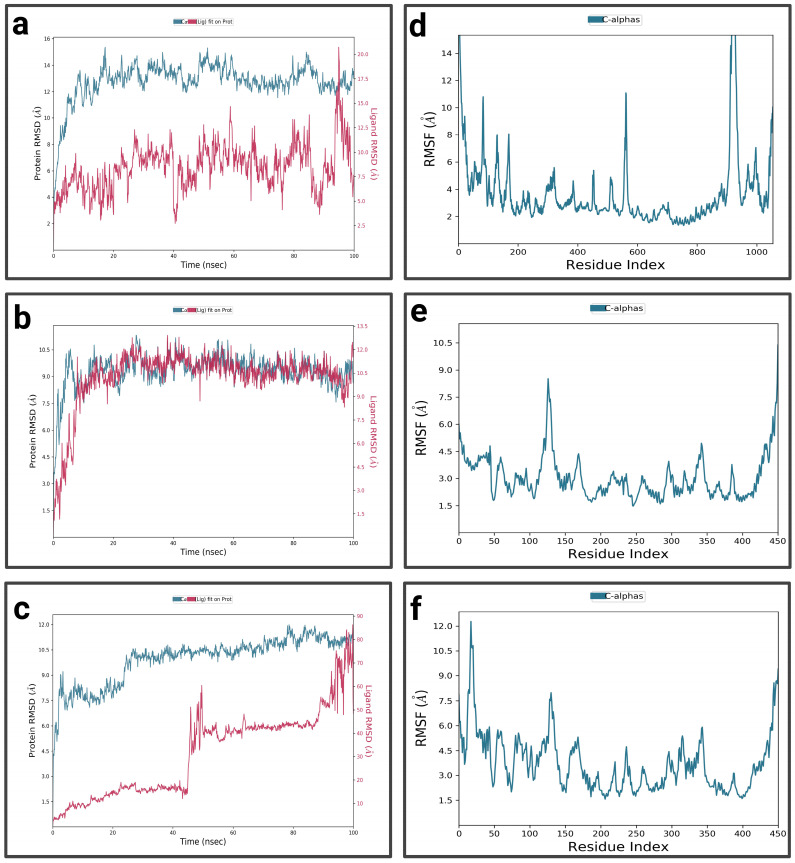
Analysis of molecular dynamics simulation: (**a**) RMSD of the P2-EGCg complex; (**b**) RMSD of the P2–chrysin complex; (**c**) RMSD of the P2–luteolin complex; (**d**) RMSF of the P2-EGCg complex; (**e**) RMSF of the P2–chrysin complex; (**f**) RMSF of the P2–luteolin complex.

For the P2-EGCg complex, the RMSD of the protein gradually increases in the first 20 ns and reaches a plateau around 13 Å, with slight fluctuations for the rest of the simulation (Figure 6a). The ligand exhibits moderate stability between 6.24 Å and 8.75 Å during the first 20 nanoseconds, indicating its binding at the P2 site with slight reorientations. Most of the residues showed significant fluctuations, ranging from 4 Å to 14 Å, suggesting flexibility in these regions (Figure 6d). Meanwhile, residues around 200 and located in the 600–800 range are characterized by moderate fluctuations of less than 4 Å, suggesting relative structural stability. For the P2–chrysin complex, the RMSD of the protein increases rapidly during the first 10 nanoseconds, reaching 5 Å, suggesting structural changes (Figure 6b). It stabilizes around 9 Å during the rest of the simulation, with small fluctuations suggesting convergence toward a relatively equilibrated protein conformation. In the first 10 ns of the simulation, the ligand exhibits an RMSD of 10 Å, indicating its binding at the P2 active site, while undergoing slight conformational adaptations within the protein pocket. The P2–chrysin profile reveals that most fluctuations are less than 3 Å, indicating structural stability (Figure 6e), with the exception of a few peaks with indices of 20, 125, and close to 400, exceeding 4.5 Å. For the P2–luteolin complex (Figure 6c), the RMSD of the protein shows fluctuations indicating structural changes. After 20 ns, it stabilizes around 10.5 Å with slight variations reflecting an equilibrated but flexible protein conformation. The ligand initially shows a low RMSD, but after 40 ns, it has a high RMSD of 40 Å, suggesting partial or total dissociation of the binding pocket, revealing that the protein–ligand interaction is unstable. The P2–luteolin profile indicates that most residues exhibit greater fluctuations exceeding 4.5 Å, which explains the increased flexibility of luteolin around residues at positions 50, 100, 120, 150–200, 300, and 350 (Figure 6f). Residues close to 200, 250, and 400 show moderate fluctuations between 3 Å and 3.5 Å.

**Figure 7:**
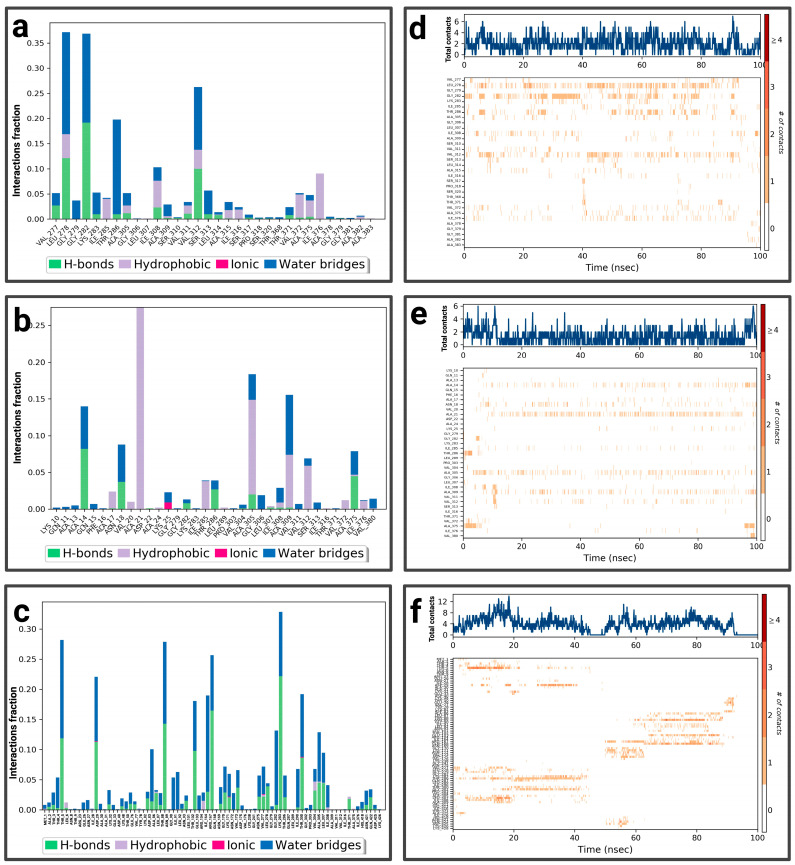
Analysis of molecular dynamics simulation: (**a**) histogram of P2-EGCg interactions; (**b**) histogram of P2–chrysin interactions; (**c**) histogram of P2–luteolin interactions; (**d**) P2-EGCg contact timeline; (**e**) P2–chrysin contact timeline; (**f**) P2–luteolin contact timeline.

For P2-EGCg, LEU 278 represents the highest fraction (0.35), reinforcing the stability of the ligand through hydrogen, hydrophobic, and hydric bonds (Figure 7a). GLY 282 represents the same fraction by establishing a hydrogen bond topped by a water bridge. THR 286 (fraction 0.20) also establishes a hydrogen bond topped by a water bridge. VAL 312 (0.25) and ILE 308 (0.10) establish a hydrophobic, hydrogen bond topped by a water bridge. The total number of contacts varies between 2 and 4 over time, suggesting relative stability. The amino acids ILE 308, LEU 278, and VAL 312 maintain persistent and regular contacts (from 2 to 6)—illustrated by a strong orange intensity—throughout the simulation (Figure 7d). Figure 7b shows the notable interactions established within the P2–chrysin complex. ALA 21 represents the highest fractional value (greater than 0.25), reinforcing the stability of the ligand through a hydrophobic interaction. ALA 305 (fraction 0.18) and ALA 309 (fraction 0.15) form hydrogen bonds, hydrophobic, topped with a water bridge. ALA 14 (fraction ≈ 0.13) establishes a hydrogen bond topped by a water bridge. Based on Figure 7e, the total number of contacts can vary between 2 and 6, with considerable stabilization after 12 ns. The amino acids ALA 21, ALA 305, ALA 309, and ALA 14 establish regular contacts (2 to 4)—illustrated by a strong orange intensity—throughout the simulation. For the P2–luteolin complex (Figure 7c), THR 286 represents the highest fractional value (exceeding 0.30), exhibiting two types of bonding (hydrogen bonding and water bonding). ASN 89 (fraction 0.27), THR 5 (≈0.28), GLN 168 (≈0.25), GLU 103 (0.175), and ALA 86 (0.10) establish the same types of interaction. ASP 29 (fraction ≈ 0.21) forms three types of bonds (hydrogen, ionic, and topped by a water bridge). According to (Figure 7f), the total number of contacts can vary between 2 and 5, with considerable stabilization after 40 ns. The amino acids ASN 89, GLN 168, GLU 103, and ALA 86 establish regular contacts (from 2 to 4), illustrated by a dark orange color. More details about the residue-level contact are provided in the Supplementary Materials.

#### 2.3.3. MD Simulations of P3 Complexes

**Figure 8:**
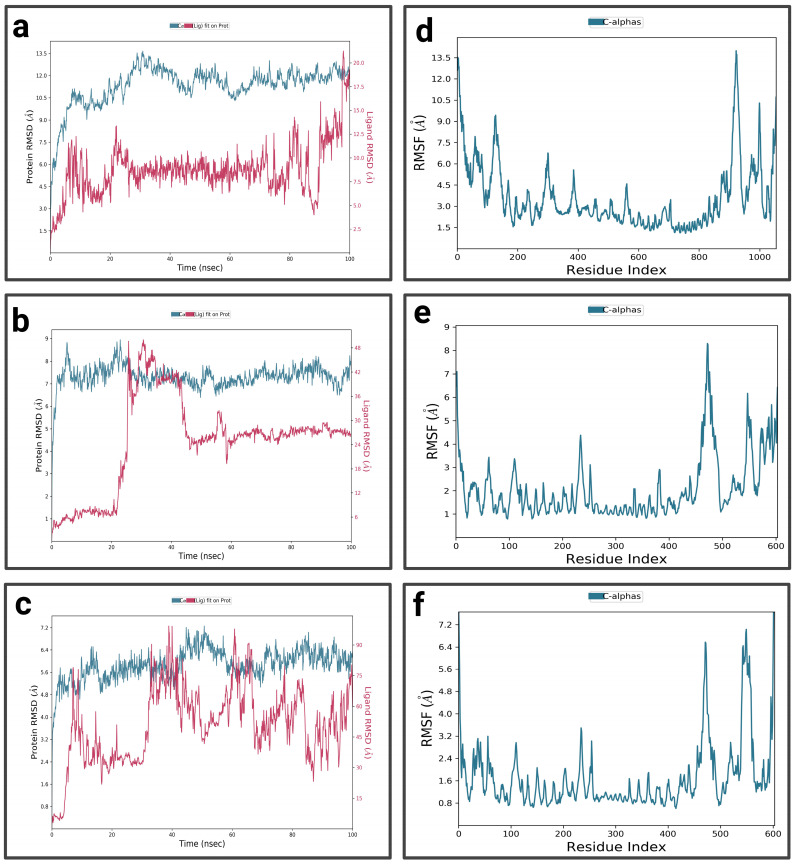
Analysis of molecular dynamics simulation: (**a**) RMSD of the P3-EGCg complex; (**b**) RMSD of the P3–chrysin complex; (**c**) RMSD of the P3–luteolin complex; (**d**) RMSF of the P3-EGCg complex; (**e**) RMSF of the P3–chrysin complex; (**f**) RMSF of the P3–luteolin complex.

In the first 10 ns, the RMSD of the complex P3-EGCg increases to around 10.5 Å, indicating structural changes (Figure 8a). It stabilizes around 11 Å with slight fluctuations, suggesting a stable but flexible conformation. In the first 20 ns and towards the end of the simulation (80 ns to 100 ns), the RMSD of the ligand (EGCg) shows fluctuations—suggesting a conformational change—and exhibits moderate stability around 8.75 Å from 25 ns to 80 ns. The RMSF profile of the P3-EGCg complex shows that most fluctuations have high RMSF values (7 Å to 13.5 Å) around index residues at around 100, 250–400, and 800–1000 (Figure 8d). Meanwhile, index residues between 400 and 800 are characterized by moderate RMSF values below 4.5 Å. For P3–chrysin, the first 5 nanoseconds of the RMSD graph showed a rapid increase, indicating structural changes (Figure 8b). It stabilizes around 6.75 Å with slight variations, suggesting a stable conformation. Chrysin stabilizes around 6 Å, indicating a stable and durable bond at the P3 active site. The P3–chrysin profile reveals that fluctuations are generally less than 3 Å for most protein residues, indicating structural stability (Figure 8e). However, a few peaks at indices 450, 550, and around 600 exceed 6 Å. For luteolin P3, during the first 5 nanoseconds, the RMSD of the protein increases to approximately 5.6 Å (Figure 8c). It stabilizes at approximately 6.4 Å, with slight variations. Between 35 ns and 40 ns, the ligand exhibits very pronounced fluctuations of 75 Å, indicating significant structural changes or suggesting total dissociation of the binding pocket, revealing that the protein–ligand interaction is unstable. For P3–luteolin, residues 475, 550, and 600 show very pronounced fluctuations exceeding 5.6 Å (Figure 8f). In contrast, residues between 100 and 400 show moderate fluctuations of less than 3.6 Å.

**Figure 9:**
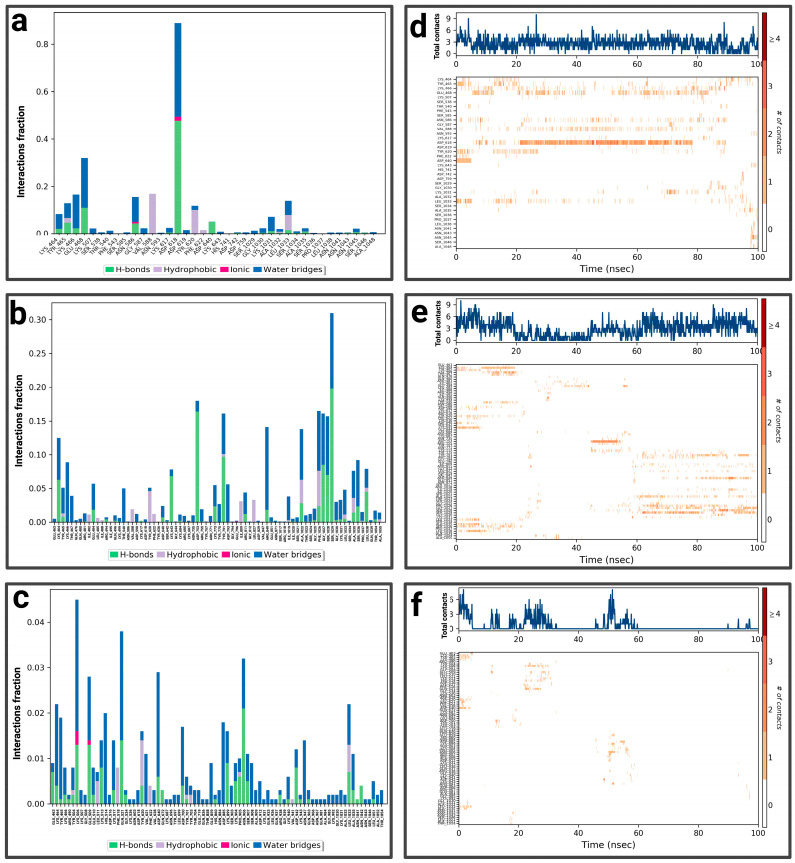
Analysis of molecular dynamics simulation: (**a**) histogram of P3-EGCg interactions; (**b**) histogram of P3–chrysin interactions; (**c**) histogram of P3–luteolin interactions; (**d**) P3-EGCg contact timeline; (**e**) P3–chrysin contact timeline; (**f**) P3–luteolin contact timeline.

For P3-EGCg, ASP 618 represents the highest fraction (0.8), mainly due to the hydrogen bond, ionic and hydric, playing a role in the stability of the ligand (Figure 9a). GLU 468 (0.3), LYS 466 (0.16), and LYS 464 (0.1) form a hydrogen bond topped by a water bridge. VAL 588 (0.17) establishes only a hydrophobic interaction. LEU 1033 (0.13) and TYR 465 (0.11) establish a hydrogen bond, hydrophobic bonds topped by a water bridge. The total number of contacts varies between 2 and 6 over time (Figure 9d). The amino acids LYS 464, TYR 465, and ASP 618 maintain frequent contacts during the simulation. For the P3–chrysin complex, SER 1029 represents the highest fractional value (greater than 0.30) formed by two types of interaction (hydrogen and hydric) (Figure 9b). ASP 753 (fraction 0.175), GLY 1027 (≈0.158), SER 1028 (0.157), and ARG 849 (≈0.13) establish two types of bonds (hydrogen topped by a water bridge). TYR 791 (fraction ≈ 0.159) and PHE 1026 (≈0.16) form three types of interactions (hydrogen bond, hydrophobic bond, and water bond). According to Figure 9e, the total number of contacts can vary between 3 and 9, with considerable stabilization after 40 ns. The amino acids SER 1029, GLY 1027, SER 1028, ARG 849, TYR 791, and PHE 1026 establish regular contacts (3 to 7)—illustrated by a dark orange color—throughout the simulation. For the P3–luteolin complex, LYS 506 represents the highest fractional value (greater than 0.04), forming three types of interaction (hydrogen, ionic, and hydric bonds) (Figure 9c). GLU 509 forms the same types of bonds with a fractional value of ≈0.027. GLN 521 (fraction ≈ 0.037), ASN 906 (≈0.033) establishes two types of bonds (hydrogen and water bonds). PRO 905 (≈0.02) forms three types of bonds (hydrogen, hydrophobic, and topped by a water bridge). ASP 885 (≈0.017) establishes a water bridge as a bond. Based on Figure 9f, the total number of contacts can vary between 3 and 6, with considerable stabilization between 20 ns and 60 ns. The amino acids PRO 905 and ASP 885 establish regular contacts (2–3)—illustrated by a dark orange color—throughout the simulation. More details about the residue-level contact are provided in the Supplementary Materials.

#### 2.3.4. MD Simulations of P4 Complexes

**Figure 10:**
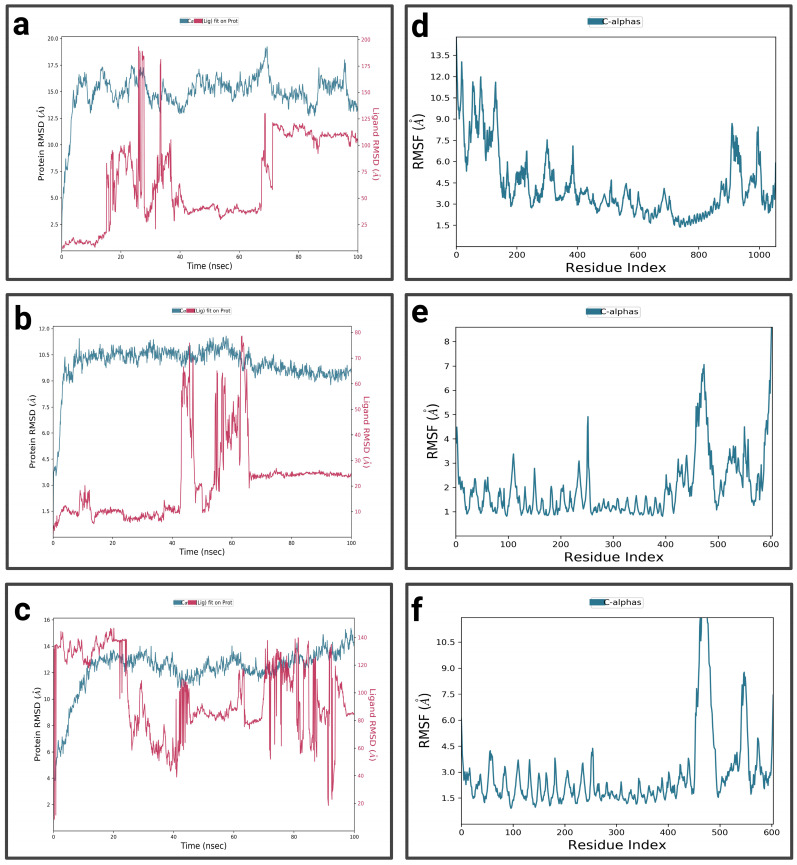
Analysis of molecular dynamics simulation: (**a**) RMSD of the P4-EGCg complex; (**b**) RMSD of the P4–chrysin complex; (**c**) RMSD of the P4–luteolin complex; (**d**) RMSF of the P4-EGCg complex; (**e**) RMSF of the P4–chrysin complex; (**f**) RMSF of the P4–luteolin complex.

For the P4-EGCg complex (Figure 10a), during the first 10 nanoseconds, the RMSD of the protein gradually increases to approximately 16 Å. It stabilizes relatively around 15 Å, with slight fluctuations. In contrast, the ligand exhibits a high RMSD (37 Å), suggesting partial or total dissociation of the binding pocket. The P4-EGCg complex is characterized by high flexibility, especially around residues 100, 200, 400, 900, and 1000, where RMSF values vary between 6 Å and 13.5 Å (Figure 10d). Residues in the range 400–800 show relative stability, with moderate RMSF values below 4.5 Å. For the P4–chrysin complex, in the first 5 ns, the RMSD of the protein increases rapidly, reaching 9 Å, and stabilizes around this value until the end of the simulation (Figure 10b). In contrast, in the first 4 ns, chrysin shows small fluctuations, suggesting a stable conformation. In the interval from 40 ns to 60 ns, the RMSD increases, after which the RMSD reaches a plateau for the rest of the simulation. The P4–chrysin profile shows that most fluctuations do not exceed 3 Å, suggesting structural stability (Figure 10e). The exceptions are the peaks around 250, 480, and 600, which exceed 4 Å. For the P4–luteolin complex, the RMSD of the protein increases gradually during the first 20 nanoseconds (Figure 10c). It stabilizes relatively around 13 Å during the rest of the simulation, with slight fluctuations. Meanwhile, the ligand exhibits pronounced fluctuations, suggesting significant conformational changes (partial or total dissociation of the P4 binding pocket). The RMSF profile of the P4–luteolin complex reveals that the residues around 450, 480, and 600 show significant fluctuations, with RMSF values greater than 8 Å (Figure 10f). The rest of the residues are characterized by moderate variations between 2 Å and 4.5 Å.

**Figure 11:**
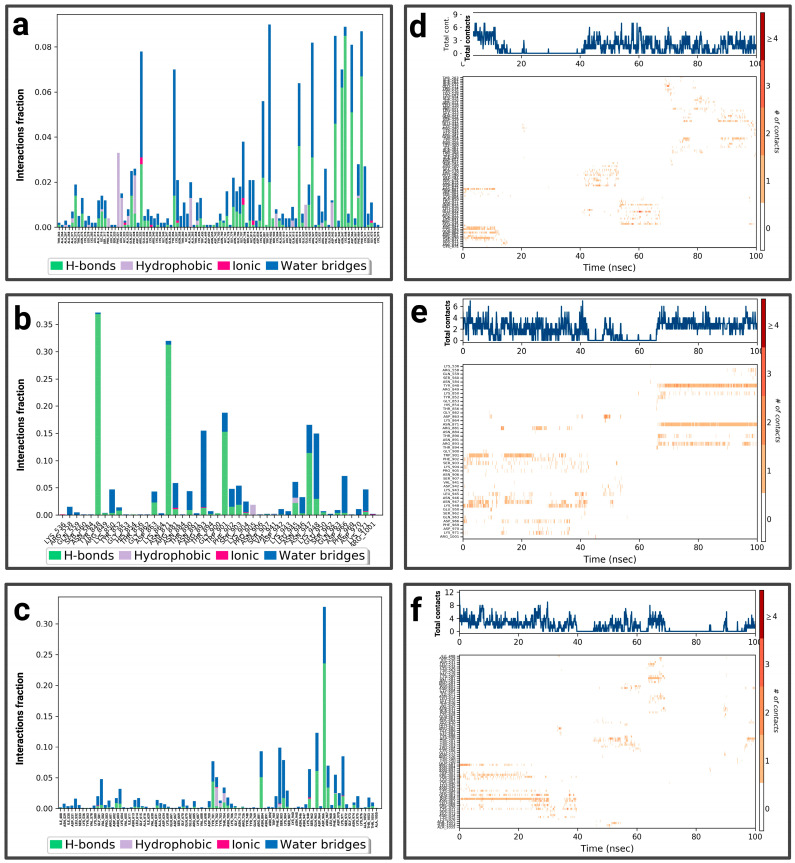
Analysis of molecular dynamics simulation: (**a**) histogram of P4-EGCg interactions; (**b**) histogram of P4–chrysin interactions; (**c**) histogram of P4–luteolin interactions; (**d**) P4-EGCg contact timeline; (**e**) P4–chrysin contact timeline; (**f**) P4–luteolin contact timeline.

For the P4-EGCg complex, ARG 881, LYS 948, ASP 970, GLU 935, and GLU 924 represent a fractional value of 0.08 by establishing a hydrogen bond topped by a water bridge (Figure 11a). TYR 356, ASN 947 (fraction 0.07), and GLN 915 (0.06) form a hydrogen and water bond. ARG 332 (0.074) establishes three types of interactions (hydrophobic, hydrogen, and hydric). The total number of contacts can vary between 2 and 6, with considerable stabilization after 40 ns. The amino acids ARG 332, TYR 356, and GLU 935 establish regular contacts (2 to 4)—illustrated by a dark orange color—throughout the simulation (Figure 11d). For the P4–chrysin complex, TYR 848 represents the highest fractional value (greater than 0.35) by forming a hydrogen bond topped by a water bridge (Figure 11b). ASN 871 (fraction ≈ 0.31), TRP 901 (≈0.187), ASN 947 (≈0.162), LYS 948 (≈0.14), LYS 850 (≈0.04), and THR 890 (≈0.05) form the same type of bond. ARG 893 (fraction ≈ 0.15) establishes three types of bonds (hydrogen, ionic, and overridden by a water bridge). According to Figure 11e, the total number of contacts can vary between 2 and 6, with considerable stabilization after 60 ns. The amino acids TYR 848, ASN 871, LYS 850, THR 890, and ARG 893 establish regular contacts (2 to 4)—illustrated by a dark orange color—throughout the simulation. For the P4–luteolin complex, ASP 966 represents the highest fractional value (greater than 0.30) by establishing a hydrogen bond and a hydric bond (Figure 11c). GLN 963 (fraction 0.125) and ASP 700/SER 903/GLY 581 (fraction ≈0.075) establish two types of bonds (hydrogen topped by a water bridge). According to Figure 11f, the total number of contacts can vary between 3 and 5. ASP 700 establishes regular contacts (2 to 4), illustrated by a dark orange color, throughout the simulation. More details about the residue-level contact are provided in the Supplementary Materials.

To sum up, RMSD and RMSF analysis reveal that chrysin and luteolin differ in their stability and conformational dynamics compared to EGCg. At the active sites (P1–P4), chrysin exhibited low RMSD values (6–7.5 Å) compared to luteolin and EGCg, which exhibited high RMSD values and notable fluctuations, suggesting increased flexibility. Analysis of protein–ligand interactions revealed that chrysin establishes strong and constant interactions (hydrogen, hydrophobic, ionic, and water bridge) that maintain the stability of the complex throughout the 100 ns simulation compared to EGCg, which establishes reduced interactions, and luteolin, which establishes moderate bonds. Therefore, the MD results confirm that chrysin showed a relatively stable complex with sites (P1–P4) compared to EGCg and luteolin.

#### 2.3.5. MM-GBSA Calculations

The MM-GBSA approach allows the binding energies of protein–ligand complexes to be calculated using the following formula [31,41]:

MMGBSA ΔG bind  = ΔG Coulomb + ΔG Covalent  + ΔG Hbond + ΔG Lipo  + ΔG Packing + ΔG SolvGB + ΔG vdW.

The ΔG (total) binding energy resulting from the interaction of ligands with the active sites of LtxA is shown in Table 2. The chrysin ligand forms a relatively stable complex, with relative free binding energies ΔG (total) of −76.77 kcal/mol, −78.43 kcal/mol, −76.28 kcal/mol, and −52.05 kcal/mol, respectively, with active sites P1, P2, P3, and P4. This strong binding affinity is due to strong lipophilic and Van der Waals interactions, as well as energy contributions from solvation. Luteolin has relative energies of −77.71 kcal/mol, −78.42 kcal/mol, −47.04 kcal/mol, and −46.04 kcal/mol with P1, P2, P3, and P4, respectively, due to lipophilic, covalent, and Van der Waals interactions, as well as energy contributions from solvation. Conversely, EGCg is characterized by less-favorable free binding energy values of −49.01 kcal/mol, −64.27 kcal/mol, −51.40 kcal/mol, and −35.21 kcal/mol with generally weaker lipophilic interactions, Van der Waals interactions, and solvation energy contributions, indicating a less-stable interaction. These results demonstrate the importance of covalent, lipophilic, and Van der Waals forces in the relative stability of the complex.

## 3. Discussion

*A. actinomycetemcomitans* is an oral Gram-negative species, facultatively anaerobic, responsible for the secretion of LtxA, and a large exotoxin belonging to the RTX family [6,42]. This toxin gives *A. actinomycetemcomitans* its virulent nature due to its ability to evade the immune system by attaching itself to either cholesterol or LFA-1 expressed on the surface of leukocytes (neutrophils, lymphocytes, and monocytes), leading to cell necrosis, allowing the bacteria to multiply and cause AP [9,15,43].

Our study was the first to investigate the potential effect of Moroccan-derived propolis compounds for the inhibition of LtxA activity in *A. actinomycetemcomitans*, specifically the simultaneous inhibition of its four active sites. Since ancient times, propolis has been considered a valuable product due to its significant therapeutic effects, except that the molecular processes involved in its functioning remain unidentified [29]. Over time, towards the end of the 15th century, researchers became interested in identifying the chemical composition of propolis, which contains more than 500 bioactive elements, including terpenes, terpenoids, polyphenols, flavonoids, phenolic compounds, and aromatic acids with several anti-apoptotic, anticancer, anti-inflammatory, antidiabetic, antibacterial, and antiviral properties [29,44]. Few studies have investigated the in vitro antimicrobial activity of propolis against periodontal and gingival microorganisms and those responsible for secondary infections [45,46]. Many studies have proven the effectiveness of propolis in reducing the depth of periodontal pockets and its antioxidant, anti-inflammatory, and antibacterial effects against various periodontal pathogens, *P. gingivalis*, *P. intermedia*, and *F. nucleatum* [47,48].

CADD relies on sophisticated computational tools to identify bioactive molecules derived from propolis that are likely to bind to protein active sites, based on simulations of ligand–protein interactions [49,50]. In this study, we adopted the Structure-Based Drug Design (SBDD) approach since the 3D structure of LtxA is available in the AlphaFold database [51]. After virtual screening of 106 molecules present in Moroccan propolis, two molecules—namely chrysin and luteolin—are considered the most promising ligands, displaying lower affinity energies, which are considered the best binding energies. In other words, low affinity energy means strong interactions, and therefore the ligand will be very effective in inhibiting the activity of the target protein [52,53] compared to epigallocatechin gallate (EGCg)—a galloylated catechin found in green tea—used as a reference, as it has been shown to have strong inhibitory activity against LtxA produced by *A. actinomycetemcomitans* [15]. At a sub-inhibitory concentration (5 μg/mL), EGCg acts as an antivirulence agent by modifying the secondary structure of LtxA, which leads to a decrease in affinity for cholesterol and LFA-1 expressed by leukocytes and an increase in affinity for bacterial membranes [15]; however, at a high concentration (50 μg/mL), EGCg inhibits bacterial growth [15]. Another study conducted by Chang and his team confirmed the previous results, showing that administration of EGCg prevents the extracellular release of LtxA and promotes its association with bacterial surfaces or with membrane vesicles, leading to decreased cytotoxicity. In contrast, cells not treated with EGCg were characterized by increased toxicity, which can lead to the death of more than 60% of immune cells [54]. Our virtual screening results are confirmed by other studies demonstrating that neoflavanoids derived from propolis have a strong binding affinity with the active sites of protein receptors in Gram (+) and (−) bacteria [52]. The optimal affinity energy of the two compounds was verified by the presence of various interactions (hydrophobic interaction, electrostatic interaction, and hydrogen bonding) that reinforce the stability of the ligand molecules at each of the four active sites [55]. Both molecules also have a good ADME-Tox profile, distinguished by very good oral bioavailability, comply with all of Lipinski’s rules, and have excellent intestinal absorption [31,33]. They have difficulty crossing the blood–brain barrier, which is desirable because our goal is to identify antibacterial molecules that do not affect the central nervous system [36]. They are characterized by low interactions with other drugs (no inhibitors and no substrates of CYP enzymes). The toxicity profile is favorable for both molecules due to total absence of genetic mutation, no hepatotoxicity, and no impact on heart rate [37]. However, chrysin is less well tolerated than luteolin due to its low maximum dose, which is lower than that of luteolin.

The calculation of the MM-GBSA parameter is considered a validation step for molecular docking [56]. The chrysin ligand forms a relatively stable complex with the four active sites due to powerful Van der Waals, lipophilic, and covalent interactions that strengthen the bond and make the complexes energetically favorable, making chrysin a promising inhibitor [41,51,57]. Luteolin establishes significant Van der Waals, lipophilic, and covalent interactions, but they are slightly weaker than those of chrysin. In contrast, EGCg establishes relatively weak bonds, indicating a less-stable interaction. These results reveal the importance of Van der Waals forces, lipophilic forces, and covalent bonds in the stability of complexes formed by chrysin and luteolin and in identifying the total binding force.

Simulating bioactive molecules over time allows us to track protein movement and distinguish the stability of complexes [41,53]. The parameters used to evaluate MD simulations include RMSD and RMSF, which are calculated to track the stability of complexes and distinguish the flexibility of residues. The number and type of interactions were evaluated during the 100 ns simulation. Analysis of the interaction timeline allows visualization of the contacts established between each protein residue and the ligand [41]. The RMSD results reveal that the protein stabilizes around high values between 9 Å and 15 Å, which is due to the flexible regions of LtxA. A study corroborates our results and indicates that large proteins, namely FKBP5, have high RMSD values between 8 Å and 10 Å, which are due to fluctuations in highly flexible and mobile regions and should not be interpreted as instability of the complexes [58]. Meanwhile, ligand stability varies depending on the complex. The results confirm that chrysin is the most promising ligand, exhibiting low RMSD values when binding to active sites (P1–P4), indicating better stability at protein active sites. Chrysin established significant hydrophobic, hydrogen, ionic, and hydrocarbon interactions, particularly with residues ASN 358, THR 274, GLN 422, and ALA 365 of site P1, as well as with ALA 21, ALA 305, ALA 309, and ALA 14 of site P2, and established the same types of interactions, with the exception of ionic bonding with SER 1029, ASP 753, GLY 1027, and SER 1028 of site P3. It mainly forms hydrogen bonds topped by water bridges, particularly with protein residues TYR 848, ASN 871, TRP 901, and ASN 947 of site P4. In contrast, the P1–luteolin complex showed fluctuations with a ligand RMSD reaching 8.25 Å, suggesting slight conformational changes at the P1 active site; however, residues THR 263, THR 368, ASN 358, THR 274, LYS 266, ALA 267, ALA 270, and ALA 365 contribute to moderate stabilization through hydrogen bonds, hydrophobic bonds, ionic bonds, and water bridges. EGCg has RMSD values reaching 18.75 Å, suggesting a rearrangement of the ligand within the P1 site. At the P2–luteolin complex level, the ligand has a high RMSD of 40 Å, suggesting partial or total dissociation of the binding pocket, revealing that the protein–ligand interaction is unstable throughout the simulation. EGCg, on the other hand, has an RMSD between 6 Å and 12.5 Å, and represents diverse interactions, including hydrogen bonds, hydrophobic bonds, and water bridges involving residues ILE 308, LEU 278, and VAL 312.

Regarding the P3–luteolin complex, the ligand showed slight fluctuations (RMSD = 6.4 Å) in the first 5 ns of the simulation, indicating stabilization maintained by hydrogen bonds and hydrophobic and water bridges involving the amino acids PRO 905 and ASP 885, but these did not last due to an increase in RMSD values reaching 75 Å, suggesting significant structural changes; however, the residues LYS 464, TYR 465, and ASP 618 contributed to moderate stabilization of EGCg through hydrogen bonds, ionic bonds, hydrophobic bonds, and water bridges. The ligands luteolin and EGCg showed pronounced fluctuations, suggesting significant conformational changes (partial or total dissociation) of the P4 binding pocket. Ligand binding to these protein residues at the LtxA active sites prevents LtxA from binding to LFA-1 or cholesterol, which inhibits leukocyte lysis [9]. Luteolin and EGCg, on the other hand, show significant variations with high RMSD values, suggesting ligand detachment or mobility at the protein’s active site [42]. RMSF analysis reveals that chrysin exerts an increased stabilizing effect on active sites by showing fluctuations generally below 3 Å, contrary to luteolin and EGCg, which are distinguished by a mixed dynamic profile with both stable and flexible regions.

Clinical trials have shown the crucial role of chrysin in reducing gastrointestinal toxicity and balancing hormones, except that pharmaceutical studies have reduced the use of chrysin as a drug due to its low intestinal permeability and low solubility [59]. The literature has reported the anticancer, antibacterial, hepatoprotective, antiradical, and antioxidant effects of this bioactive molecule, demonstrating its strong interaction with the active sites of an enzyme responsible for the conversion of tumor necrosis factor α (TNF-α) and superoxide dismutase (SOD) [60]. Li-Ping Sun and his colleagues examined nine samples of Chinese propolis, identifying chrysin as the primary active compound. This active ingredient has been shown in vitro and in vivo to have anticancer properties by inhibiting HDAC8, which is capable of slowing tumor proliferation and inducing differentiation in human breast cells [61]. Researchers have demonstrated the crucial role of this flavonoid in preventing hormone-dependent tumors by inhibiting aromatase, which is essential in the conversion of androgens into estrogens [62]. There is one exception of a single study that showed that human consumption of propolis over a period of twenty days did not actually have an effect; however, this is explained by constant or unchanged levels of testosterone in urine, which contradicts in vitro research [63].

Zhang and his colleagues confirmed the antibacterial activity of Chinese red propolis—which consists mainly of chrysin and 14 other bioactive compounds, namely pinobanksin-3-acetate and pinobanksin—against *S. aureus* and Methicillin-resistant *S. aureus* (MRSA) by inhibiting bacterial proliferation through deterioration of the bacterial wall and membrane of both strains and by inhibiting bacterial self-destruction and virulence [64]. Other studies have focused on the resistance of *Acinetobacter baumannii* to antibiotics, particularly colistin. Researchers have shown that the use of chrysin at a minimum inhibitory concentration (MIC) exceeding 128 µg/mL has no effect on bacterial growth. However, combining or associating chrysin with conventional antimicrobials is a very effective alternative for inhibiting bacterial resistance by preventing biofilm formation and modifying the bacterial membrane [65]. Experimental studies demonstrate the binding of chrysin to an important drug-binding site on human serum albumin with an association constant (Ka = 2.7 × 10^5^ M^−1^), which prevents the binding of other ligand molecules. The binding of chrysin causes structural changes in the protein structure and leads to changes in protein conformation and stability [66,67].

One study demonstrated that chrysin and its derivatives, obtained through microwave-assisted O-alkylation, exhibited antimicrobial activity against *P. aeruginosa*, *Klebsiella pneumoniae*, and Methicillin-resistant *S. aureus* [68]. In other research, scientists have combined this natural flavonoid, chrysin, with methoxypolyethylene glycols to optimize its solubility in water [69]. They observed an improvement in antibacterial and antifungal activity, while its anticancer and antioxidant properties remained unchanged but effective [69]. Alipour and colleagues used chrysin-based scaffolds to evaluate their effectiveness in treating internal dental problems (endodontics) [70]. These scaffolds have successfully inhibited the bacteria responsible for dental infections, namely *A. baumannii*, *S. aureus*, *P. aeruginosa*, and *Enterococcus faecalis* [70]. They protect against pulp infections, activate the proliferation of pulp stem cells, and play a key role in bone healing by promoting biomineralization [70]. Otherwise, many research teams have focused on the second molecule in our study, luteolin, which has demonstrated an effective role in inhibiting bacterial growth, including *E. coli*, *S. aureus*, and *P. aeruginosa*, in association with antibiotics [71]. A study conducted by Lo and his colleagues highlighted the low bioavailability of luteolin. To solve this problem, the researchers synthesized derivatives of this flavonoid, which showed improved anticancer activity (breast and colon cancer), antioxidant activity, and improved bioavailability [72]. Another study has shown the antibacterial efficacy of this bioactive flavonoid against *Trueperella pyogenes* by increasing the permeability of the bacterial wall and membrane and disrupting protein synthesis [73]. Similarly, one study has shown that luteolin derived from *Lophatherum gracile* acts as an antibacterial agent against multidrug-resistant *E. coli* by destroying the integrity of the cell wall and membrane, reducing ATP production, and preventing the expression of genes resistant to sulfonamides and quinolones [74].

An in vitro study shows that luteolin is capable of inhibiting the binding of the RBD domain expressed by the SARS-CoV-2 spike protein and the ACE2 ligand. Luteolin prevents this interaction with a value (IC50 = 0.61 mM). The *in silico* study demonstrates that luteolin establishes strong bonds, particularly with the amino acids involved in stabilizing the complex [75]. Research shows that luteolin inhibits the growth of *S. aureus* and Methicillin-resistant *S. aureus* with a minimum inhibitory concentration of (MIC = 31.25 µg/mL) and a bacteriostatic activity MBC/MIC ratio > 4.0. This inhibition is due to luteolin’s ability to form a stable complex with the virulent enzyme sortase A [76].

## 4. Materials and Methods

### 4.1. Protein Structure Retrieval

The 3D structure of the LtxA protein (UniProt: P16462) was obtained from the AlphaFold database (UniProt P16462) (DeepMind, London, United Kingdom; European Molecular Biology Laboratory — European Bioinformatics Institute, Hinxton, United Kingdom). It has a high predicted Local Distance Difference Test (pLDDT) score of 72.12, indicating good protein region modeling and, therefore, reliable prediction [77,78].

### 4.2. Preparation of Proteins

The preparation was performed using AutoDock tools version 1.5.7 (The Scripps Research Institute, La Jolla, CA, USA) by removing water molecules, adding Kollman atomic charges and polar hydrogens, and then saving them in PDBQT format [79]. LtxA has 4 active sites, namely P1 and P2, located in the N-terminal domain of the α-helix center; P3, which is identified in an area between the fifth and sixth RTX repeats; and finally, P4, which is found between the fourteenth repeat of the RTX domain [9]. A grid was developed for each active site, and the grid frame was inserted to fully encompass the active site: P1(x = −61.34; y = −6.827; z = 47.335); P2 (x = −40.198; y = −17.063; z = 44.815); P3 (x = 6.037; y = 15.833; z = −17.786); P4 (x = 29.409; y = −3.769; z = −44.053) [9,31].

### 4.3. Data Collection and Ligand Preparation

A database containing 106 molecules derived from Moroccan propolis—known for its numerous properties, particularly antibacterial and antifungal—was employed [80]. This database served to identify ligands that attach simultaneously to the 4 active sites (P1, P2, P3, and P4) of LtxA [9]. PubChem (National Center for Biotechnology Information, Bethesda, MD, USA), provided the 3D structures of ligands in SDF format, which were converted to PDB format using Open Babel 3.1.1 [16,17]. The preparation of the ligands was performed using AutoDock Tools version 1.5.7 by adding Gasteiger charges, polar hydrogens, and then saving in PDBQT format [31,79].

### 4.4. Molecular Docking

Molecular docking is the Swiss-knife of CADD approaches that makes it easier to predict the ideal interaction between a ligand and its receptor and accelerates the search for new drugs [81]. A virtual multitarget screening was conducted by a Perl script using the AutoDock Vina software 1.2.5 (The Scripps Research Institute, La Jolla, CA, USA), which generates a massive amount of results in a very short time, offering a calculation speed up to one hundred times faster than AutoDock and, thus, providing a more precise and accurate prediction of binding modes [31,82]. The docking was performed with grid boxes of dimension 40 × 40 × 40 Å^3^ centered on each of the aforementioned active sites, and the exhaustiveness parameter was set to 20. The best position of the ligand, distinguished by a better binding energy, was retained for the continuation of the analysis [51].

### 4.5. Visualization and Analysis

Ligands were selected based on their binding affinity measured in kcal/mol. Those with a binding affinity less than or equal to that of EGCG were visualized using Discovery Studio [31].

### 4.6. Prediction of ADME-Tox Properties

ADMET properties play a crucial role in drug design [83]. A precise prediction of these properties facilitates the selection or identification of the most effective molecules for treatments, thereby avoiding any failures during clinical trials and allowing for a better understanding of the fate of the drug within the human body [83,84]. In this study, these properties were predicted by an online server called pkCSM ADMET (https://biosig.lab.uq.edu.au/pkcsm/) (accessed on 29 December 2025) [83,85]; by entering the SMILES structures of the most promising ligands, it was possible to evaluate Lipinski’s rules, properties such as skin permeability, intestinal absorption, blood–brain barrier permeability, CYP2D6/CYP3A4 substrates and inhibitors, total clearance, AMES toxicity, maximum tolerated dose, hERG I/II inhibitors, and hepatotoxicity [83,85,86].

### 4.7. Molecular Dynamics Simulations

Ligands with a better binding affinity to the protein target LtxA and meeting ADME-Tox criteria were subjected to a molecular dynamics (MD) simulation to visualize structural adjustments and the flexibility of the ligand–protein complexes over time [87,88]. In this study, the Desmond software (D. E. Shaw Research, New York, NY, USA) included in Schrodinger Maestro (Schrödinger, LLC, New York, NY, USA), version 2019-4 (https://www.schrodinger.com/platform/products/desmond/) (accessed on 7 November 2025) [89] was used to perform the MD simulations [89].

Simulations make it possible to calculate the interactions established in a protein–ligand complex and predict the binding state of the ligand in a physiological environment while integrating Newton’s equation [90,91]. The Maestro protein preparation assistant improves protein and ligand structures by removing poor shapes, unwanted overlaps, and bad contacts [90]. The simulation system was developed using the System Builder tool, which determines the TIP3P solvent model (transferable 3-point intermolecular interaction potential), the orthorhombic simulation box, and the OPLS_2005 force field [89,90]. The electrical neutrality of the model is ensured by the addition of counter ions and sodium chloride at a concentration of 0.15M [89,90]. Before neutralization, the total charge in the systems is +20, and the number of Na^+^/Cl^−^ ions added is +70/−90 for P1–chrysin; +68/−88 for P2–chrysin; +84/−86 for P3–chrysin; +84/−86 for P4–chrysin; +70/−90 for P1–luteolin; +68/−88 for P2–luteolin; +84/−86 for P3–luteolin; and +84/−86 for P4–luteolin.

Simulations must be performed under physiological conditions at a temperature of 300 kelvins (K), a pressure of 1 atmosphere (atm), and in an NPT system. Trajectories were recorded every 100 picoseconds (ps) [89,92]. The stability of the complexes was determined by the root mean square deviation (RMSD), root mean square fluctuation (RMSF), and the contacts established between the protein and the ligand [89].

### 4.8. MM-GBSA Analysis

In order to evaluate the free energy (ΔG binding) of binding of a protein–ligand complex, the MM-GBSA method was used [93,94]. The calculations were performed using the prime module of the Schrodinger Maestro software, version 2019-4 (https://www.schrodinger.com/platform/products/desmond/) (accessed on 7 November 2025) which employs both the OPLS3 force field and the VSGB solvation model to simulate the interactions between atoms while considering the effect of water on the molecules [31,93,94,95].

## 5. Conclusions

*A. actinomycetemcomitans* is strongly associated with periodontitis and oral infections. The antibacterial properties of propolis, a naturally occurring resinous material that honeybees gather from plant exudates, have been extensively researched. Propolis could not only be a viable natural adjuvant in periodontal therapy because it has potent antibacterial and antibiofilm properties against *A. actinomycetemcomitans* and other species, but could also be a source of many pure compounds with marked antimicrobial potency. Hence, the present in silico study identified chrysin and luteolin, two molecules derived from Moroccan propolis, as potential LtxA inhibitors due to their high binding affinities to its four active sites. These bioactive molecules have a satisfactory ADME-Tox profile, which explains their therapeutic potential against AP, except that chrysin has shown greater stability, making it a more promising candidate. However, in vitro and in vivo studies are still needed to confirm our promising results.

## Figures and Tables

**Table 1:** In silico prediction of the ADME-Tox properties of chrysin and luteolin.

		Chrysin	Luteolin
Lipinski’s Rules		Yes	Yes
Absorption and distribution	Skin Permeability (log Kp)	−2.739	−2.735
	Water Solubility (log mol/L)	−3.538	−3.094
	Intestinal Absorption (human) (%)	93.761	81.13
	Caco2 Permeability (log Papp in 10^−6^ cm/s)	0.945	0.096
	Blood–Brain Barrier (Log BB)	0.047	−0.907
Metabolism	CYP2D6/CYP3A4 Substrates (Yes/No)	No	No
	CYP2D6/CYP3A4 Inhibitors (Yes/No)	No	No
Excretion and toxicity	Total Clearance (log ml/min/kg)	0.405	0.495
	AMES Toxicity	No	No
	Max. Tolerated Dose (human) (log mg/kg/day)	0.016	0.499
	hERG I and II Inhibitors	No	No
	Hepatotoxicity	No	No

**Table 2:** MMGBSA binding energy of the complex formed by EGCg, chrysin, or luteolin and active site LtxA (P1, P2, P3, and P4). (H-bond: hydrogen bond energy, Lipo: lipophilic energy, Solv GB: generalized Born electrostatic solvation energy, vdW: Van der Waals energy).

Complex	ΔG Binding (Total)	ΔGbind Coulomb	ΔGbind Hbond	ΔGbind Covalent	ΔGbind Lipo	ΔGbind Solv GB	ΔGbind vdW	ΔGbind Packing
P1-EGCg	−49.01	−18.56	−0.51	1.93	−21.74	13.57	−23.70	0.0
P1–chrysin	−76.77	−6.45	−0.45	1.305	−47.58	11.18	−34.78	0.0
P1–luteolin	−77.71	−19.03	−0.49	1.75	−42.47	15.62	−33.08	0.0
P2-EGCg	−64.27	−15.65	−0.52	1.60	−35.81	10.05	−23.93	0.0
P2–chrysin	−78.43	−12.34	−0.673	0.258	−46.21	11.4	−30.9	0.0
P2–luteolin	−78.42	−12.33	−0.67	0.257	−46.21	11.40	−30.87	0.0
P3-EGCg	−51.40	−10.50	−0.11	0.28	−31.54	19.11	−28.64	0.0
P3–chrysin	−76.28	−20.89	−0.59	1.99	−43.26	22.83	−36.35	0.0
P3–luteolin	−47.04	−20.31	−2.73	1.49	−32.63	20.1	−28.65	0.0
P4-EGCg	−35.21	−17.99	−0.97	2.55	−14.38	16.41	−20.83	0.0
P4–chrysin	−52.05	−21.95	−0.49	1.137	−25.21	17.12	−22.65	0.0
P4–luteolin	−46.04	−25.12	−1.95	5.00	−16.00	19.2	−27.15	0.0

## Data Availability

The original contributions presented in this study are included in the article. Further inquiries can be requested from the corresponding author.

## Supplementary Materials

The following supporting information can be downloaded at https://www.mdpi.com/article/10.3390/ph19010115/s1, Table S1: Detailed analysis of residues involved in the interaction of EGCg, chrysin, and luteolin with the P1, P2, P3, and P4 binding sites of LtxA.
