# Supplementary material for: Chrysin and Luteolin from Moroccan Propolis to Prevent Aggressive Periodontitis Caused by Aggregatibacter actinomycetemcomitans Leukotoxin: A Computer-Aided Drug Design Approach
Source: Pharmaceuticals (Basel). 2026 Jan 8;19(1):115. doi: 10.3390/ph19010115 (PMC12844875; doi:10.3390/ph19010115)
Supplement: Supplementary file 1 [file pharmaceuticals-19-00115-s001.zip › pharmaceuticals-4029684-supplementary.pdf]

Supplementary Materials

Chrysin and Luteolin from Moroccan Propolis to Prevent Aggressive Periodontitis Caused by *Aggregatibacter actinomycetemcomitans* Leukotoxin: A Computer-Aided Drug Design Approach

Doha EL Meskini [1], Fettouma Chraa [1], Jihane Touhtouh [2], Mouna Ouadghiri [1], Monica Gallo [3,*], Abdelhakim Bouyahya [4] and Tarik Aanniz [1,*]

1 Medical Biotechnology Laboratory (MedBiotech), Bioinova Research Center, Medical and Pharmacy School, Mohammed V University in Rabat, Rabat 10100, Morocco; elmeskinidoha2@gmail.com (D.E.M.); fettoumachraa04@gmail.com (F.C.); m.ouadghiri@um5r.ac.ma (M.O.)

2 Laboratory of Natural Resources and Environment, Polydisciplinary Faculty of Taza, Sidi Mohamed Ben Abdellah University of Fez, Taza 30050, Morocco; jihanetou97@gmail.com

3 Department of Molecular Medicine and Medical Biotechnology, University of Naples Federico II, Via Pansini 5, 80131 Naples, Italy

4 Laboratory of Human Pathologies Biology, Faculty of Sciences, Mohammed Vth University in Rabat, Rabat 10100, Morocco; a.bouyahya@um5r.ac.ma

* Correspondence: mongallo@unina.it (M.G.); t.aanniz@um5r.ac.ma (T.A.)

Table S1. Detailed analysis of residues involved in the interaction of EGCg, chrysin, and luteolin with the P1, P2, P3, and P4 binding sites of LtxA

**Complex**	**Details**
**P1 site**	
**P1–EGCg**	GLU 80 represents the highest fraction percentage (1.4) formed by a water bridge and a hydrogen bond, revealing its significant role in stabilizing the ligand. LEU 87 (0.8), GLY 95 (0.4), GLU 112, and ASN 93 with a fraction of 0.1 establish the same type of interactions. TYR 52 (with a fraction of 0.39) forms a hydrogen bond overridden by a water bridge.
**P1–chrysin**	For chrysin, interactions were established with ASN 358 representing the highest fractional percentage (0.37), formed by a hydrogen and a water bond. THR 274 (fraction ≈ 0.25), HIS 418 (≈ 0.15), and LEU 256 (≈0.05) form the same type of interactions. GLN 422 reaches a fractional value around 0.152 formed by a hydrogen bond and ionic bond which are overcome by a water bridge. ALA 267 (fraction≈ 0.05) formed by a hydrogen bond, ionic topped by a water bridge. ALA 365, VAL 415, and ALA 270 form hydrophobic bonds, topped by a water bridge with fractions of ≈ 0.149, 0.10, and 0.07 respectively.
**P1–luteolin**	SER 411 represents the highest interaction percentage (greater than 0.4) and THR 263 (fraction 0.35) formed by a hydrogen bond topped by a water bridge. and THR 368, with respective fraction indices of and 0.09, establish the same type of interactions. ASN 358 (fraction 0.39), THR 274 (fraction 0.3), LYS 266 (fraction 0.2), HIS 418 (fraction 0.13) establish three types of hydrogen and ionic bonds topped by a water bridge. ILE 361 (fraction 0.12), ALA 414 (fraction 0.1), ALA 267 (fraction 0.09), and ALA 362 (fraction 0.08)

	form three types of hydrogen interactions, hydrophobic and hydric bonds. ALA 270 (fraction 0.12) and ALA 365 (fraction 0.15) form two types of hydrophobic interactions topped by a water bridge.
**P2 site**	
**P2–EGCg**	LEU 278 represents the highest fraction (0.35), reinforcing the stability of the ligand through hydrogen, hydrophobic, and hydric bonds. GLY 282 represents the same fraction by establishing a hydrogen bond topped by a water bridge. THR 286 (fraction 0.20) also establishes a hydrogen bond topped by a water bridge. VAL 312 (0.25) and ILE 308 (0.10) establish a hydrophobic, hydrogen bond topped by a water bridge, while ILE 376 (0.075) forms a hydrophobic bond.
**P2–chrysin**	ALA 21 represents the highest fractional value (greater than 0.25), reinforcing the stability of the ligand through a hydrophobic interaction. ALA 305 (fraction 0.18), ALA 309 (fraction 0.15), and ALA 376 (fraction 0.07) form hydrogen bonds, hydrophobic topped with a water bridge. ALA 14 (fraction ≈ 0.13), ASN 18 (fraction 0.08), and THR 286 (fraction 0.029) establish a hydrogen bond topped by a water bridge. VAL 312 (fraction 0.06) and ILE 285 (fraction 0.03) have two different types of bonds (a hydrophobic bond topped by a water bridge). LYS 25 (fraction ≈0.02) forms two distinct types of bonds (an ionic bond topped by a water bridge).
**P2–luteolin**	THR 286 represents the highest fractional value (exceeding 0.30), exhibiting two types of bonding (hydrogen bonding and water bonding). ASN 89 (fraction 0.27), THR 5 (≈0.28), GLN 168 (≈ 0.25), ARG 167 (0.187), GLU 103 (0.175), LYS 283 (0.125), and ALA 86 (0.10) establish the same types of interaction. ASP 29 (fraction ≈0.21) and GLY 302 (fraction ≈0.187) form three types of bonds (hydrogen and ionic topped by a water bridge. LEU 307 and ILE 08 form bonds (hydrogen, hydrophobic topped by a water bridge) with fraction values of ≈ 0.12 and 0.08, respectively.
**P3 site**	
**P3–EGCg**	ASP 618 represents the highest fraction (0.8), mainly due to hydrogen bond, ionic and hydric, playing a role in the stability of the ligand. ASN 585 (fraction 0.15) establishes the same types of interactions. GLU 468 (0.3), LYS 466 (0.16), and LYS 464 (0.1) form a hydrogen bond topped by a water bridge. VAL 588 (0.17) establishes only a hydrophobic interaction, TYR 620 (0.1) forms a hydrophobic bond topped by a water bridge, while LEU 1033 (0.13) and TYR 465 (0.11) establish a hydrogen bond, hydrophobic bonds topped by a water bridge.
**P3–chrysin**	SER 1029 represents the highest fraction value (greater than 0.30) formed by two types of interaction (hydrogen and hydric). ASP 753 (fraction 0.175), GLY 1027 (≈ 0.158), SER 1028 (0.157), ARG 849 (≈ 0.13), LYS 464 (0.125), SER 1036 (0.08),-LYS 643 (0.075), and GLU 484 (≈ 0.059) establish two types of bonds (hydrogen topped by a water bridge). TYR 791 (fraction ≈0.159), PHE 1026 (≈0.16), ALA 1022 (≈ 0.13), ALA 1035 (0.075), LEU 1038 (0.075), and LEU 1033 (≈ 0.05); form three types of interactions (hydrogen bond, hydrophobic bond, and water bond). LYS 466 (fraction 0.08), ASP 793 (≈ 0.059), THR 540 (0.05), and TYR 465 (≈ 0.05) form only a water bridge. TYR 620 (≈ 0.05) establishes a hydrophobic bond topped by a water bridge.
**P3–luteolin**	LYS 506 represents the highest fractional value (greater than 0.04), forming three types of interaction (hydrogen, ionic, and hydric bonds). GLU 509

	forms the same types of bonds with a fractional value of ≈ 0.027. GLN 521 (fraction ≈ 0.037), ASN 906 (≈ 0.033), ASP 640 (≈ 0.028), LYS 464 (≈ 0.023), TYR 465 (≈ 0.018), ASP 700 (≈0.016), LYS 899 (≈0.015), ASP 942 (≈ 0.012), ALA 1035 (≈ 0.01), LYS 513, and LYS 517 fraction (≈ 0.014) establish two types of bonds (hydrogen and hydric). LEU 1033 (fraction ≈ 0.024), TYR 620 (≈ 0.016), and PRO 905 (≈ 0.02) form three types of bonds (hydrogen, hydrophobic topped by a water bridge). HIS 514 (fraction ≈0.02), ASP 885 (≈ 0.017), and ASN 947 (≈ 0.014) establishes a water bridge as a bond.
**P4 site**	
**P4–EGCg**	ARG 881, LYS 948, ASP 970, GLU 935, and GLU 924 represents a fraction value of 0.08 by establishing a hydrogen bond topped by a water bridge ASP 966 (fraction 0.075), TYR 356, and ASN 947 (fraction 0.07), GLN 915 (0.06), ASP 834 (0.055), LYS 931/GLN 327 (0.025), ASP 798/ALA 778 (0.02) form a hydrogen and water bond. ARG 332 (0.074), GLU 796 (0.035), PHE 969 (0.025), and LYS 357 (0.021) establishes three types of interactions (hydrophobic, hydrogen and hydric). PHE 321 (0.03) forms only a hydrophobic interaction. ILE 361 (0.02) forms a hydrophobic bond, surmounted by a water bridge.
**P4–chrysin**	TYR 848 represents the highest fractional value (greater than 0.35) by forming a hydrogen bond topped by a water bridge. ASN 871 (fraction ≈ 0.31), TRP 901 (≈ 0.187), ASN 947 (≈ 0.162), LYS 948 (≈ 0.14), ASP 966 (≈ 0.075), SER 903 (≈ 0.05), LYS 850 (≈ 0.04), GLY 900 (≈ 0.04), ASP 863 (≈ 0.04), and THR 890 (≈ 0.05) form the same type of bond. ARG 893 (fraction ≈0.15); ARG 881 (≈0.06), LYS 971 (≈0.03), and LYS 904 (≈ 0.025) establishes three types of bonds (hydrogen, ionic overridden by a water bridge). LEU 945 (≈ 0.06) establishes three types of bonds (hydrogen, hydrophobic, and hydric).
**P4–luteolin**	ASP 966 represents the highest fractional value (greater than 0.30) by establishing a hydrogen bond and a hydric bond. GLN 963 (fraction 0.125); ARG 881 (≈ 0.09), PHE 902 (≈0.10), LYS 971 (≈0.08), ASP 700/ SER 903/GLY 581 (fraction ≈0.075), ASN 967 (≈ 0.06), and PHE 969 (≈ 0.053) establish two types of bonds (hydrogen topped by a water bridge). PHE 902 (fraction ≈0.10), LYS 971 (≈ 0.08), and LYS 948 (≈ 0.054) forms three types of bonds (hydrogen, ionic, and hydric).
